# Effect of consuming novel foods consisting high oleic canola oil, barley β-glucan, and DHA on cardiovascular disease risk in humans: the CONFIDENCE (Canola Oil and Fibre with DHA Enhanced) study – protocol for a randomized controlled trial

**DOI:** 10.1186/s13063-015-1014-5

**Published:** 2015-10-31

**Authors:** Vanu R. Ramprasath, Sijo J. Thandapilly, Shuo Yang, Anjalika Abraham, Peter J. H. Jones, Nancy Ames

**Affiliations:** Richardson Centre for Functional Foods and Nutraceuticals, University of Manitoba, 196 Innovation Drive, Winnipeg, MB R3T 2N2 Canada; Department of Human Nutritional Sciences, University of Manitoba, Winnipeg, MB Canada; Agriculture and Agri-Food Canada, Winnipeg, MB Canada

**Keywords:** Canola oil, Barley, β-glucan, DHA, Fiber, Cardiovascular disease, Metabolic syndrome, CONFIDENCE trial

## Abstract

**Background:**

Metabolic syndrome (MetS) has been identified as a major contributor to the development of cardiovascular disease (CVD). Current recommendations for dietary management of people with MetS involve quantitative and qualitative modifications of food intake, such as high consumption of vegetables, fruits, and whole grain foods. The results from our previous human trials revealed the potential of the dietary components high-oleic acid canola oil (HOCO)-docosahexaenoic acid (DHA) and high molecular weight barley β-glucan individually in managing CVD risk factors. Foods with a combination of HOCO-DHA and barley β-glucan have never been tested for their effects on CVD risk. The objective is to determine the effects of consuming novel foods HOCO-DHA, and barley β-glucan on managing CVD risk factors in people with MetS.

**Methods/Design:**

We are conducting a randomized, single-blind crossover trial with four treatment phases of 28 days each separated by a 4-week washout interval. Participants (n=35) will be provided with weight-maintaining, healthy balanced diet recommendations according to their energy requirements during the intervention periods. Participants will receive muffins and cookies as treatment foods in a random order and will consume at least one meal per day at the research center under supervision. The four treatments include muffins and cookies consisting of (1) all-purpose flour and HOCO-DHA (50 g/day); (2) barley flour (4.36 g/day of β-glucan) and a blend of sunflower oil, safflower oil, and butter as control oil (50 g/day); (3) barley flour (4.36 g/day of β-glucan) and HOCO-DHA (50 g/day; dosage of DHA would be 3 g/day); and (4) all-purpose flour and control oil (50 g/day). At the beginning and end of each phase, we will evaluate anthropometrics; systolic and diastolic blood pressure; blood lipid profile; low-density lipoprotein subfractions and particle size; 10-year Framingham CVD risk score; inflammatory status; and plasma and red blood cell fatty acid profiles, fecal microbiome, and body composition by dual-energy X-ray absorptiometry.

**Conclusion:**

Cholesterol synthesis will also be studied, using a stable isotope approach. The proposed study will lead to innovation of novel food products, which may result in improvement in the overall cardiovascular health of humans.

**Trial registration:**

Clinical trials.gov identifier: NCT02091583. Date of registration: 12 March 2014.

## Background

Metabolic syndrome (MetS), comprising a spectrum of chronic disease risk factors that include abdominal obesity, dyslipidemia, hypertension, and elevated fasting plasma glucose, has been identified as a major contributor to the development of cardiovascular disease (CVD) [1, 2]. In North America, 34 % of the adult population are affected by MetS, and MetS occurrence is even higher in other parts of the world [3]. Accordingly, MetS has become a significant public health concern and a burden on the global health care system, as this disorder is associated with increased morbidity, mortality, and related health care costs.

Current recommendations for dietary management in people with high risk for MetS involve quantitative and qualitative modifications in food intake, such as higher consumption of vegetables, fruits and whole grain food components with their inherent bioactive composition. More specifically, the presence of Mediterranean dietary components [4, 5], amounts of long chain n-3 fatty acids [6, 7], types and amounts of carbohydrates and dietary fiber content, are some dietary elements reported to show remarkable positive effects in the management of MetS. Important components include high-oleic acid canola oil (HOCO), barley β-glucan, docosahexaenoic acid (DHA).

Canola oil is derived from canola seed, which is obtained from a bright yellow flowering plant belonging to the Brassicaceae family [8]. Canola oil is considered the third-largest vegetable oil by sales volume after palm and soybean oil [9]. Canola’s nutritional profile includes a low level (7 %) of saturated fatty acids; significant amounts of monounsaturated fatty acids (MUFAs) and polyunsaturated fatty acids (PUFAs), with 61 % oleic acid, 21 % linoleic acid, and 11 % α-linolenic acid [10]; plant sterols (PSs) (0.53–0.97 %); and tocopherols (700–1200 ppm) [8]. Canola oil has proven efficacy in preventing and/or managing chronic ailments, including dyslipidemia, insulin resistance, and inflammation [11, 12]. As a result, in 2006, the U.S. Food and Drug Administration (FDA) approved a qualified health claim for canola oil suggesting that canola oil may help reduce the risk of CVD when substituted for saturated fat in the diet [8].

MUFAs, one of the components of canola oil, help reduce CVD risk by lowering blood pressure, blood total cholesterol (TC), and low-density lipoprotein cholesterol (LDL-C) levels; inhibiting LDL-C oxidation [13–16]; and improving glycemic control [17–19]. Oleic acid–enriched canola oil was developed to enhance the health benefits of canola oil related to CVD risk reduction. Its health benefits were compared with regular canola oil in humans in a randomized crossover human trial [12]. In that trial, consumption of HOCO for 28 days was shown to lead to reductions in TC and LDL-C levels and LDL-C/high-density lipoprotein cholesterol (HDL-C) values, as compared with regular canola oil and a control diet without HOCO [12]. The results of a recently completed multicenter randomized controlled trial done at our laboratory validated these findings. In that study, the serum triglycerides (TGs) and 10-year Framingham risk scores, TC/HDL-C and TG/HDL-C ratios, and systolic blood pressure were significantly reduced, and HDL was increased, after consumption of HOCO-DHA compared with regular canola and control diets [20–23].

Consumption of fiber has been indicated to reduce the mortality rate in high-risk CVD populations [24]. β-glucan is a polysaccharide found mainly in yeast, mushrooms, oats, and barley in the form of fiber [25]. β-glucan fibers are located mainly in the endosperm cell walls of cereals. Among cereals, barley and oats are the richest sources of β-glucan, containing 4.0–7.0 % and 2.2–7.8 % wt/wt of β-glucan, respectively, compared with other cereals, such as rye and wheat [26]. Similarly to canola oil, β-glucan has gained significant attention among nutritional researchers because it has been shown to have potentially health-promoting effects in animal and human studies [25, 27]. Consequently, in 1997, the FDA approved a health claim regarding β-glucan from whole oats for reducing the risk of heart disease [28, 29]. More recently, the European Food Safety Authority and Health Canada approved similar health claims for foods containing oats or barley β-glucan [30, 31].

In agreement with the previous studies, we also demonstrated the beneficial effects of high molecular weight β-glucan in healthy adults in a recent randomized, controlled-diet, crossover trial (n = 35). The results showed that consumption of 3 g or 5 g per day of high molecular weight barley β-glucan is highly effective in reducing TC and LDL cholesterol in adults with moderate hypercholesterolemia [32].

Long-chain PUFAs such as eicosapentaenoic acid (EPA) and DHA from fish and marine source oils, including algal oil, have commercially emerged as dietary supplements due to their health benefits. Long-chain fatty acids, including DHA, were shown to lower TG and increase serum HDL-C concentrations [33]. DHA is also capable of acting as an antiatherogenic factor by inhibiting inflammatory processes and thereby inhibiting plaque formation [34]. On the basis of the vast amount of knowledge accumulated over the years, the American Heart Association has recommended the use of n-3 fatty acids, including DHA, for secondary prevention of cardiovascular events in people with documented coronary artery disease.

Considering the fact that CVD is a multifactorial disease, the dietary approaches that combine different bioactives with enhanced health benefits have recently been well elucidated. For instance, a portfolio containing a low-fat vegetarian diet enriched by the inclusion of PSs, soy protein, viscous fiber, and tree nuts has been shown through our collaborations to markedly reduce LDL-C concentrations to an extent comparable in magnitude with reductions achieved using first-generation statins [35, 36]. Likewise, a large body of research has demonstrated the synergetic effects attained by introducing two or more food bioactive components into the diet to achieve maximal health benefits through multimechanistic effects [37–39]. Accordingly, combining highly bioactive components such as HOCO, barley β-glucan, and DHA is expected to produce great synergistic and pleotropic beneficial effects on CVD risks factors compared with the individual biological actions listed above.

The overall objective of the present study is to test novel foods containing the most effective bioactives, including n-3 fatty acid–enriched dietary oil high in MUFAs and soluble dietary fiber, with aims of managing CVD risk factors in people with MetS and testing the efficacy and safety of these dietary components in humans.

## Methods/Design

### Design of the human intervention trial

**Fig. 1:**
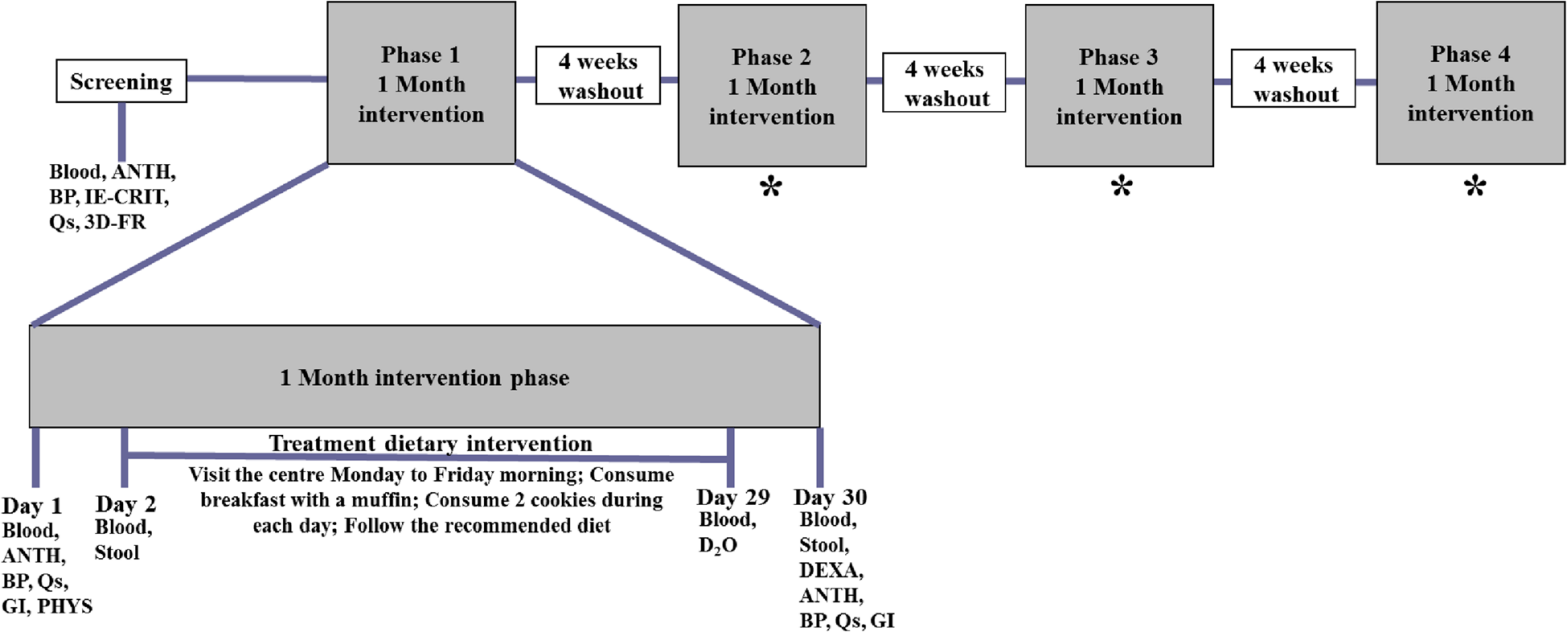
Schematic representation of the experimental protocol. *Procedures are the same as in phase 1. *Blood* 12-h fasted blood collection, *ANTH* anthropometric measurements, *BP* blood pressure, *IE-CRIT* inclusion and exclusion criteria, *Qs* questionnaires (fish and seafood consumption, concomitant medications, adverse events, physical activity), *3D-FR* 3-day food record collection, *DEXA* dual-energy X-ray absorptiometry, *PHYS* physician examination, *GI* gastrointestinal questionnaires, *STOOL* stool sample collection, *D*_*2*_ *O* deuterated water

A randomized, single-blind, crossover trial will be conducted at the Clinical Nutrition Research Unit at the Richardson Centre for Functional Foods and Nutraceuticals (RCFFN), University of Manitoba, Winnipeg, MB, Canada. The study design will consist of four phases with 28 days per phase, and each phase will be separated by 4-week washout periods (Fig. 1). Participants will consume at least one of three daily meals under supervision, along with the treatment foods during weekdays from days 2 to 29 of each phase. Treatment muffins and cookies for the weekend will be packed and provided to participants to take home on Fridays. The other meals will be purchased by the participants from grocery stores on their own. Almost half of the treatments will be consumed under the supervision of a clinical coordinator to ensure optimal compliance. Participants will be instructed to consume no alcoholic or caffeinated beverages. Participants will be instructed not to consume more than one serving of fish or seafood products per month during the study. A 3-day food record will be collected before the study and after each treatment phase. Participants will be strongly recommended to maintain consistency in their physical activities during the experimental period. Compliance with the treatment products will be determined from checklists and by measuring the amount of leftover food if any in the containers returned by the participants the following day.

Diets will be planned for every participant according to individual energy requirements and will be nutritionally adequate. Basal energy requirement will be estimated using the Mifflin-St Jeor equation [40]. The basal energy requirements will be multiplied by an activity factor of 1.7 to supply the additional energy needs of mildly to moderately active healthy adults. During the study period, body weight will be monitored. If any of the participants gain or lose weight during the first week, adjustments to their estimated energy requirements will be made. Participants will be given recommendations to follow a typical Western diet that meets the Canadian Dietary reference intake. The nutrient content of the participant’s diet, based on the 3-day food record, will be analyzed using Food Processor Nutrition Analysis software (ESHA Research, Salem, OR, USA) to ensure the intake of macro- and micronutrients and compliance with the protocol. Treatments will be isocalorically incorporated into muffins and cookies and consumed at breakfast, in the evening, as snacks, and with supper. Recruited participants will be randomly assigned to one of the treatment sequences using a Latin square design. Participants will be required to consume one muffin along with their breakfast and two cookies during their evening snack and dinner, respectively.

The four treatments are as follows: (1) muffins and cookies containing all-purpose flour and 50 g/day HOCO-DHA (85 %/15 %; DHA oil consists of 40 % DHA) (DHA dosage 3 g/day); (2) muffins and cookies containing barley flour with 4.36 g/day of high molecular weight barley β-glucan and 50 g/day of a blend of sunflower oil, safflower oil, and butter as a control oil; (3) muffins and cookies containing a combination of barley flour with 4.36 g/day, high molecular weight barley β-glucan, and 50 g/day of HOCO-DHA; and (4) muffins and cookies containing all-purpose flour and 50 g/day of control oil. The control oil used in the study represents a typical Western diet fat intake as a control treatment composed largely of saturated fat with substantial levels of n-6 linoleic acid common in current North American dietary intakes. Dosages for HOCO-DHA and barley β-glucan were determined on the basis of previous clinical studies done by our research group [20, 21, 32]. Muffin and cookie recipes were developed in Agriculture and Agri-Food Canada laboratories at the RCFFN metabolic kitchen. Barley grain (cultivar CDC Rattan) was provided by the Alberta Barley Commission and milled into whole grain flour by the Canadian International Grains Institute. All-purpose flour was obtained from local supermarket. HOCO was provided by Richardson Oilseed Limited. life’s DHA oil was purchased from DSM Nutritional Products (Ayr, ON, Canada). Macronutrients, including protein, carbohydrates, and fiber content of the foods, were analyzed at the Agriculture and Agri-Food Canada laboratories, RCFFN, Winnipeg, Canada. Total fat and fatty acid profiles of the foods were measured at the RCFFN laboratories (Winnipeg, MB, Canada). The muffin flavors include vanilla and spice, and the cookies were made in lemon, ginger, and chocolate flavors. The flavors of the muffins and cookies will be provided in rotation with equal numbers of days per flavor during each treatment period. The nutrient compositions of the muffins and cookies for the different treatments of the study are depicted in Tables 1 and 2, respectively. The macronutrient contents of treatment foods will be similar between different treatments, and also there are no differences in nutritional values between different flavors of muffins or cookies.

**Table 1:** Details and nutritional composition of the study muffins

Nutritional component measurements	Control flour + HOCO-DHA	Barley flour + control oil	Barley flour + HOCO-DHA	Control flour + control oil
Weight (g)	113	108	109	112
Energy (kcal/kJ)^a^	379/1586	383/1602	383/1602	379/1586
Total carbohydrates (g)^a^	33	36.59	36.59	32.65
Total proteins (g)^a^	8	9	9	8
Total fiber (g)^a^	0.36	5.77	5.77	0.36
β-glucan (g)^a^	0	2.18	2.18	0
Total fat (% weight)	24	23	23	23
SFA (%)	10.42	35.36	11.24	33.34
MUFA (%)	66.21	28.38	64.03	31.05
PUFA (%)	23.37	36.25	24.73	35.61
n-3 PUFA (%)	6.57	0.66	6.45	0.49
n-6 PUFA (%)	16.80	35.60	18.28	35.12

*DHA* docosahexaenoic acid, *HOCO* high-oleic acid canola oil, *MUFA* monounsaturated fatty acid, *PUFA* polyunsaturated fatty acid, *SFA* saturated fatty acid

^a^Values were calculated based on values of the ingredients. Muffins were made with two different flavors: vanilla and spice. Values presented are the average of both the flavors, and there were no major differences between flavors for the measurements

**Table 2:** Details and nutritional composition of the study cookies

Nutritional component measurements	Control flour + HOCO-DHA	Barley flour + control oil	Barley flour + HOCO-DHA	Control flour + control oil
Weight (g)	52	50	52	53
Energy (kcal/kJ)^a^	213/891	221/924	221/924	213/891
Total carbohydrates (g)^a^	23	25	25	23
Total proteins (g)^a^	4	4	4	4
Total fiber (g)^a^	0.46	3.17	3.17	0.46
Beta glucan (g)^a^	0	1.09	1.09	0
Total fat (% weight)	24	24	23	24
SFA (%)	12.50	35.39	12.58	33.75
MUFA (%)	63.37	28.95	64.23	31.08
PUFA (%)	24.13	35.67	23.20	35.16
n-3 PUFA (%)	6.44	0.61	6.28	0.48
n-6 PUFA (%)	17.53	34.91	16.91	34.69

*DHA* docosahexaenoic acid, *HOCO* high-oleic acid canola oil, *MUFA* monounsaturated fatty acid, *PUFA* polyunsaturated fatty acid, *SFA* saturated fatty acid

^a^Values were calculated based on the values of the ingredients. Cookies were made with three different flavors: cocoa, ginger, and lemon. Values presented are the average of the flavors, and there were no major differences between flavors for the measurements

### Participant selection

Thirty-five male and female participants with MetS will be enrolled in the study. They will be recruited using advertisements in local media, including newspapers, radio, and the RCFFN official website, as well as on university campuses. We will also recruit from our preexisting database of participants. As well, to present information about the nature of the study and procedures, sessions will be held at the RCFFN during the recruitment period, and potential study participants will be invited to these sessions. Potential participants will undergo a screening where blood pressure and anthropometric measurements will be performed and 10-ml fasting blood samples will be collected to test for lipid profiles. Eligible participants will be asked to provide written informed consent to participate in the study. The trial was approved by the biomedical research ethics committee at the University of Manitoba (B2014:029).

### Inclusion criteria

Participants aged 18–70 years will be recruited. The participants should be slightly overweight with a body mass index >25 kg/m^2^ and waist circumference >94 cm for men and >80 cm for women in accordance with the International Diabetic Federation MetS criteria for waist circumference. In addition, they have to be deemed to be otherwise healthy by the study physician. Additionally, participants should also meet at least two of the following criteria: (1) TG >1.7 mmol/L, (2) HDL-C <1 mmol/L for males and <1.3 mmol/L for females, (3) fasting glucose >5.6 mmol/L, (4) LDL-C >2.7 mmol/L, and (5) blood pressure >130 mmHg for systolic and >85 mmHg for diastolic.

### Exclusion criteria

Participants taking lipid-lowering medications or nutritional supplements known to affect blood lipids, or who have any dietary restrictions that would prevent them from consuming the study diet for 28 days during each phase, will be excluded. Participants with a current or past history of any diseases and disorders that could interfere with fat absorption will be excluded. Individuals with hypertension having systolic blood pressure >150 mmHg or diastolic blood pressure >100 mmHg will be excluded from the study. Participants planning to become pregnant during the study period will be excluded. Smokers and people consuming more than one alcoholic drink/day or who have a history of alcoholism or drug dependence cannot be included in the study. Use of any experimental medication within 1 month before screening or as concomitant medication is also an exclusion criterion.

### Sample collection

Twelve-hour fasting blood samples will be collected on days 1, 2, 29, and 30 of each intervention phase. Participants will be advised to be fasted for 12 h and not to consume any alcoholic beverage for at least 48 h before blood collection. Participants will also be instructed to refrain from engaging in intense physical activity (aerobic, spinning, and running) for at least 24 h before each visit. On days of blood collection, a checklist with questions on 12-h diet, 48-h alcohol, and 24-h physical activity will be administered and data recorded to determine participant compliance.

Blood samples will be centrifuged at 3000 rpm for 20 minutes at 4 °C; aliquoted to yield serum, plasma, and red blood cells (RBC); and then stored at −80 °C until analyses. To obtain the plasma lipid profile, average values of days 1 and 2 will be considered as baseline and average values of days 29 and 30 will be considered as the endpoint of each treatment phase. In addition, the influence of these diets on gastrointestinal microbial diversity in the current study population will be measured. For this analysis, stool samples (four or five scoops totaling 4 g) will be collected before and after each intervention phase (days 2 and 30) of the trial. Stool sample collection kits, including containers, will be provided for the participants to collect their stool samples. However, it is optional for the participants to provide the stool samples, and they may still participate if they choose not to provide stool samples.

### Stable isotope tracer intake

To assess cholesterol fractional synthesis rate (FSR), participants will be asked to consume deuterated water (D_2_O) at the end of each phase. On day 29, 0.7 g of D_2_O per kilogram of estimated body water will be given orally before breakfast as a tracer to measure cholesterol FSR over 24 h. Fasting blood will be obtained on days 29 and 30.

### Clinical data collection

Whole-body dual-energy X-ray absorptiometry (DEXA) (Prodigy Advance; GE Healthcare Lunar, Madison, WI, USA) will be conducted to determine changes in body fat composition during the last week of each phase. Blood pressure, body weight, and waist circumference will be monitored at the beginning and end of each treatment period. An automated blood pressure device will be used to measure blood pressure. Participants will be advised to rest quietly throughout the measurements. Blood pressure will be measured four times at 2-minute intervals. The first measurement will be ignored, and the last three measurements will be averaged to determine systolic and diastolic blood pressures. Gastrointestinal tolerability questionnaires will be completed by participants at the beginning and end of each intervention period. The 10-year Framingham CVD risk score will be calculated for each participant during each phase [41].

### Analytical methodology

Plasma samples will be analyzed for total lipid profiles, including TC, HDL-C, and TG levels, as well as for glucose levels, using the automated enzymatic methods on a VITROS 350 Chemistry System (Ortho Clinical Diagnostics, Markham, ON, Canada). LDL-C values will be calculated using the Friedewald equation [42]. Subfractions and particle sizes of LDL-C after each treatment intervention will be determined by the proprietary Lipoprint Lipoprotein Subfractions Testing System (Quantimetrix, Redondo Beach, CA, USA).

Inflammatory markers such as plasma C-reactive protein and serum amyloid A levels will be measured with commercially available enzyme-linked immunosorbent assay (ELISA) kits. Plasma cytokines, specifically interleukin (IL)-1, IL-6, IL-8, IL-10, and tumor necrosis factor-α, will be measured by multiplex human ELISA. Plasma soluble adhesion molecules such as soluble vascular cell adhesion molecule 1, soluble intercellular adhesion molecule 1, and soluble P-selectin and E-selectin will also be measured with commercially available human ELISA kits. Apolipoproteins A1, B, and E will be analyzed with commercially available ELISA kits.

Fasting plasma insulin concentrations will be determined using an ELISA kit. Insulin homeostasis modeling assessment will be used as an estimate for percentage β-cell function and insulin resistance.

#### Plasma and red blood cell fatty acid analyses

Plasma and RBC total lipids will be extracted using the Folch method [43], involving chloroform-methanol (2:1 vol/vol) containing 0.01 % 3,5-di-*tert*-4-butylhydroxytoluene (Sigma-Aldrich, Oakville, ON, Canada) and heptadecanoic acid as an internal standard (Sigma-Aldrich). Extracted fatty acids will be methylated with methanolic HCl. Fatty acid methyl esters will be separated on a SUPELCOWAX 10 capillary gas chromatography column (30 m × 0.25 mm with 0.25-mm film thickness; Supelco/Sigma-Aldrich, Bellefonte, PA, USA) using a gas chromatograph equipped with a flame ionization detector (Bruker 430; Bruker Daltonics, Billerica, MA, USA). Individual fatty acids will be identified by comparison with known standards (Nu-Chek Prep, Elysian, MN, USA). Individual fatty acid levels will be calculated according to the peak area relative to the total area and expressed as a percentage of total fatty acids.

Concentrations of isotope-labeled cholesterol in circulatory cholesterol will be measured over 24 h. In this study, we will measure the deuterium enrichment into RBC cholesterol to calculate FSR of whole-body cholesterol. The initial 24-h interval of D_2_O uptake is considered to be the optimal period for measuring synthesis [44]. The analytical methods for isotope analyses are as follows. For determining cholesterol FSR (%/day), RBC from day 29 and 30 will be saponified with freshly prepared KOH-methanol at 100 °C for 1 h, and the sterol fraction will be extracted with petroleum diethyl ether. RBC cholesterol deuterium enrichment will be quantified using a gas chromatography-pyrolysis-isotope ratio mass spectrometry (Delta V Pulse Isotope Ratio Mass Spectrometer; Thermo Fisher Scientific, Bremen, Germany). Hydrogen gas reduced from water will be analyzed for deuterium enrichment against Vienna Standard Mean Ocean Water [45].

#### Microbiome analysis

Bacterial DNA from the fecal samples will be extracted using the ZR Fecal DNA MiniPrep kit (Zymo Research, Irvine, CA, USA), and DNA concentration and quality will be determined using a NanoDrop 2000c spectrophotometer (NanoDrop Products/Thermo Fisher Scientific, Wilmington, DE, USA). Gut microbial composition will be analyzed by next-generation Illumina-based sequencing (Illumina, San Diego, CA, USA). Briefly, the V3-V4 region of the 16S rRNA gene will be targeted for PCR amplification as described by Fadrosh et al. [46]. PCR products will then be purified using ZR-96 DNA Clean-up Kit (Zymo Research) and added to the MiSeq Reagent Kit V3 (300 cycles; Illumina) for sequencing reactions on a MiSeq platform. The paired-end Illumina fastq files will then be analyzed using the QIIME software package [47]. Principal coordinate analysis will be performed to evaluate the distance matrices by generating two-dimensional plots using PRIMER v6 software (PRIMER-E, Ivybridge, UK).

### Sample size calculation and statistical analysis

Sample size was determined to detect an anticipated difference in LDL-C level of 7.4 % (0.43 mmol/L) due to HOCO consumption, compared with control, with a standard deviation estimate of 0.13 mmol/L. The α and power were taken as 0.05 and 0.8, respectively. A sample size of 35 participants was therefore calculated, with a target of 28 participants, taking into account the block size and the estimated 20 % premature withdrawal rate of participants from the protocol, given the existing level of difficulty in completing the protocol elements.

Statistical analysis will be performed using SAS statistical software (SAS Institute, Cary, NC, USA). Baseline and endpoint measurements will be compared using the analysis of variance (ANOVA) model for determination of treatment effects. The results will be expressed as means with standard errors. The normality of distribution of data will be determined with the Shapiro–Wilk test, and the non-normal variables will be normalized before comparison with other treatment by log transformation. The effects of dietary treatments will be examined using mixed-model ANOVA with diet, sequence, phase, and center as fixed factors and subject as a random factor within the model. Statistical significance will be set at *p* < 0.05 for all analyses. Significance between different dietary effects will be examined with the Bonferroni adjustment for multiple comparisons.

### Adverse events and concomitant medications

No adverse events due to the study treatments are expected, based on the previous studies [20] and because the experimental components are foods. Adverse events, if any, will be monitored and will be reported in the case report form. All serious adverse events will be reported to sponsors and the institutional ethics board of the University of Manitoba. Serious adverse events will be brought to the attention of the study physician immediately for assignment of attribution. All prescription and over-the-counter medications either used at the start of the trial or initiated during the trial will be recorded.

### Conditions for withdrawal from the study

The following events are to be considered sufficient reason for discontinuing a participant from the study: poor compliance (consuming <70 % of the given treatment foods and/or dietary guidelines) or noncompliance by the participant; developing condition(s) or consumption of fish, supplements, or medications as specified under the exclusion criteria; and presenting with an adverse event or any other medical situation where continuation of the study’s treatment would compromise the participant's health.

### Confidentiality of data

All the data collected for this study will remain confidential in accordance with the Personal Health Information Act of Manitoba, as applicable. However, the clinical research team members involved will know the identity of the participants. The researchers may know the identity of the participants if they are requested to respond to questions during the informed consent process. All research team members will respect the concept of confidentiality at all times.

Each participant will be assigned a unique code so that all personal information, including questionnaire information, will be confidential. Full names and other identifying information will not be revealed, unless required by law and/or for the applicable research ethics board review or for auditing purposes. The identity of the participants will not be revealed in any published data or in presentation of the information obtained as a result of this study. Medical records containing the identity of the participant will be treated as confidential.

### Data handling and storage

All data collected will be collated and secured in a locked filing cabinet in a locked office at the RCFFN and made available only to the study investigators and study coordinators for research purposes. The data will include patient history information, which will be recorded when enrolling the participants in the trial. The purpose of accessing the data will be restricted to creation of final results tables and statistical interpretation. The data will not be used for any other purposes.

### Participant feedback

The primary outcomes, including the 10-year Framingham CVD risk score and blood lipid profile results, will be given to the study participants when the analyses are completed. Participants will receive a sealed and confidential letter that states their individual results for the parameters measured for study purposes, along with the mean values obtained for the entire study population. The letter will be sent by the principal investigator at the RCFFN to the mailing address listed on the personal information form that participants fill out before enrollment in the study.

## Discussion

Considering the huge health impact of dietary portfolio intervention approaches as highly efficient nutritional strategies for the modification of CVD risk factors, combination of food components and bioactives are getting tremendous attention as an alternative strategy to manage CVD risk factors. We anticipate that, taken together, the foods containing both HOCO-DHA and barley-derived high molecular weight β-glucan will possess a superior biological action due to their differential mechanistic effects, compared with the individual components, against CVD risk factors.

In the current proposal, combining these potent groups of food bioactives, such as HOCO-DHA and β-glucan, to achieve parallel multiplication of biological action and to reduce the CVD events substantially is of paramount clinical significance. This study will be the first investigation of the cholesterol-lowering effect of a combination of HOCO-DHA and barley β-glucan. The proposed study will help to improve understanding of the metabolic mechanisms underpinning the health benefits associated with consumption of foods consisting of HOCO-DHA and barley β-glucan. The proposed research will not only support the improvement of the cardiovascular health of Canadians but also increase the marketability of our canola-barley products internationally. The anticipated outcomes of the proposed study are expected to have a high probability of impacting the health and wellness of Canadians because (1) a significant proportion of the population has MetS and (2) scientific data generated by well-designed, unbiased human clinical trials are required to properly substantiate health effects of a combination of HOCO-DHA and barley β-glucan.

## Trial status

The trial is registered at ClinicalTrials.gov (NCT02091583). Muffins and cookies required for the study have been developed and analyzed for their nutritional content. The clinical intervention of the study was initiated in January 2015, and recruitment and randomization of participants, along with the study dietary interventions, are ongoing.

## Abbreviations

ANOVA: Analysis of variance
ANTH: Anthropometric measurements
BP: Blood pressure
CVD: Cardiovascular disease
DEXA: Dual-energy X-ray absorptiometry
3D-FR: 3-day food record collection
DHA: Docosahexaenoic acid
D_2_O: Deuterated water
ELISA: Enzyme-linked immunosorbent assay
EPA: Eicosapentaenoic acid
FDA: U.S. Food and Drug Administration
GI: Gastrointestinal questionnaires
FSR: Fractional synthesis rate
HDL-C: High-density lipoprotein cholesterol
HOCO: High-oleic acid canola oil
IE-CRIT: Inclusion and exclusion criteria
IL: Interleukin
LDL-C: Low-density lipoprotein cholesterol
MetS: Metabolic syndrome
MUFA: Monounsaturated fatty acid
PS: Plant sterol
PUFA: Polyunsaturated fatty acid
Qs: Questionnaires (fish and sea food consumption, concomitant medications, adverse events, physical activity)
RBC: Red blood cells
RCFFN: Richardson Centre for Functional Foods and Nutraceuticals
SFA: Saturated fatty acid
TC: Total cholesterol
TG: Triglyceride
